# Factors associated with COVID-19 vaccination behaviour in Latvian population: cross-sectional study

**DOI:** 10.1080/21642850.2022.2085108

**Published:** 2022-06-07

**Authors:** Sanita Šuriņa, Kristīne Mārtinsone, Gatis Upesleja, Viktorija Perepjolkina

**Affiliations:** a Department of Health Psychology and Pedagogy, Rīgas Stradiņš University, Riga, Latvia; b Faculty of Communication, Rīgas Stradiņš University, Riga, Latvia

**Keywords:** COVID-19 vaccination behaviour, fear of COVID-19, institutional trust, perceived vulnerability, perceived social support

## Abstract

Vaccination is the most effective way of limiting the spread of COVID-19. However, despite the proven effectiveness and safety of vaccines, there is resistance in society and the course of vaccination is slow. The aim of this study was to identify the factors associated with COVID-19 vaccination behaviour.

**Methods:** The data originate from a representative sample of Latvian residents (*N*  = 1017) taken in September 2021. The data were analysed using Chi-squared test, Mann–Whitney test, Spearman's rank correlation coefficient, Kruskal Wallis test, and Binary Logistic regression analysis.

**Results:** The results of the study reveal several factors that are associated with COVID-19 vaccination behaviour. These factors are a higher level of education, motivation for protecting oneself against illness, for promoting collective immunity, protecting relatives and other people from infection, as well as motivation for vaccination in order to keep a job or continue studies, and institutional trust. On the other hand, perceived social support from relatives is negatively associated with vaccination behaviour.

**Conclusion:** A higher level of education and confidence in evidence-based information on COVID-19, provided by official sources of information, is the key factor in deciding whether to protect oneself from serious illness or to make a choice to promote collective immunity and protect other people. The need for vaccination in order to continue working and/or learning is also an essential motive for vaccination. On the other hand, the belief that, if necessary, it is possible to receive support from relatives may be a delaying factor in the behaviour of vaccination.

## Introduction

According to the World Health Organisation (WHO, 2021), vaccination against COVID-19 is the most effective way to reduce the spread of the disease. The first vaccines against COVID-19 were already available in some countries at the end of 2020 (Parka, 2022). Vaccines were available to Latvian residents in January 2021 (LSM.lv, 2022). Studies point to the unquestionable effectiveness and safety of vaccines (Pormohammad et al., 2021). However, despite the calls of health experts, vaccination against COVID-19 has been slow in many parts of the world (Ritchie et al., 2022), including Latvia (COVID-19, 2022). The public demand for COVID-19 vaccines was relatively high in May and June 2021, when the first vaccines became available. The next wave of active vaccination was from September to November 2021, when many workplaces required a vaccination certificate to continue working and/or studying. In total, by April 2022, 69% of the Latvian population had completed the vaccination course (NVD, 2022).

According to studies, vaccination behaviour is linked to a number of factors that inhibit or promote vaccination behaviour, such as institutional trust, fear of COVID-19, perceived vulnerability, perceived social support, individual motives for vaccination, educational level, age, etc. (AlShurman, Khan, Mac, Majeed, & Butt, 2021; Al-Amer et al., 2022).

Vaccination is one of the types of preventive behaviour. Preventive behaviour can be defined as any action recommended by healthcare professionals to prevent disease (Rad et al., 2021). In order to explore factors associated with COVID-19 vaccination behaviour this study sets up an integrative model based on the Social capital (SC) and Protection motivation theory (PMT) framework. The inclusion of these theories in a single model, can help to explain the vaccination behaviour, taking into account the interaction between psycho-emotional and socio-demographic factors.

SC can be defined as a resource that an individual acquires in relations with other individuals or social groups (Ehsan, Klaas, Bastianen, & Spini, 2019) and can be described in three ways. Bonding SC describes an individual's relationship within homogenous groups. Bridging SC describes an individual's relationship within heterogeneous groups. Linking SC describes institutionalised relationships (Šuriņa, Ozerska, Maķevica, Zariņa, & Grate, 2021). Based on the literature analysis (Al-Amer et al., 2022), the model of this study includes SC factors, such as perceived social support from relatives (bonding SC), acquaintances (bridging SC) and institutional trust (linking SC).

The framework of the PMT allows for the assessment of internal, individual psychological factors related to vaccination behaviour (Tong, He, Wu, Dang, & Chen, 2021; Wang et al., 2021), which are operationalised as the perceived vulnerability and fear of disease, as part of this study. In addition to the factors of SC and PMT, the integrative model also includes key factors associated with vaccination behaviour, such as individual motives for vaccination (Marco-Franco, Pita-Barros, Vivas-Orts, González-de-Julián, & Vivas-Consuelo, 2021; Reinders et al., 2020; Rieger, 2020) and socio-demographic factors (age and level of education) (Al-Amer et al., 2022). The following defines the variables included in the integrative model and describes the relationship between these variables.

Any behaviour is based on motivation that gives the behaviour direction and purpose. According to studies, one of the most significant factors, associated with the behaviour of vaccination, is the need to be vaccinated in order to protect oneself from serious illness (Reinders et al., 2020), as well as the desire to protect one’s relatives and others (Giubilini, Savulescu, & Wilkinson, 2020; Machida et al., 2021; Rieger, 2020). The motive for protecting other people from illness is very important given the dangers of COVID-19 for the elderly, people with chronic diseases, or weakened immunity, etc. (Giubilini et al., 2020). Nevertheless, as already mentioned above, largely the public's attitude toward vaccines is ambiguous (Ruiz & Bell, 2021; Simione, Vagni, Gnagnarella, Bersani, & Pajardi, 2021). Therefore, in several countries (Euronews, 2022), including in Latvia (MK, 2021) vaccination against COVID-19 is employers’ requirement for individual professionals. As a result, the need to keep a job or continue studies may be a motive for vaccination (Marco-Franco et al., 2021; Palm, Bolsen, & Kingsland, 2021; Riva, Paladino, Paleari, & Belingheri, 2021).

The motive for engaging in action to protect own health is closely linked to the cognitive and emotional assessment of the potential threat, which is described in PMT (Tong et al., 2021). The perceived vulnerability within the PMT is explained as a component of the threat assessment (the other component is the perceived severity of the threat) and can be defined as a subjective assessment of the risk of the disease, the individual's belief in susceptibility to the disease and its effects (He, Chen, Kong, & Liu, 2020). The higher the probability of infection, the more likely the individual will be vaccinated to avoid the disease (Lin, Yen, Chang, & Wang, 2021; Tong et al., 2021; Wang et al., 2021). At the same time, the results of several studies show that, in the case of vaccination against COVID-19, perceived vulnerability may not be associated with behaviour (Hromatko, Tonković, & Vranic, 2021; Lau et al., 2010). There may be a number of reasons for this, such as dispositional optimism (Kapoor & Singhal, 2021), an increase in awareness and knowledge of COVID-19 (Moline et al., 2021) or specific issues related to the measurement of perceived vulnerabilities (Van Der Pligt, 1998). The perceived vulnerability, within PMT, is associated with fear, namely the higher perceived vulnerability, or the confidence in the likelihood of infection, the more pronounced fear of the disease (Adunlin et al., 2020).

Fear is defined as unpleasant feelings that arise when an individual perceives threatening stimuli (Cori et al., 2021). Studies, conducted during the COVID-19 pandemic, show a positive association between fear of COVID-19 and vaccination behaviour (Sekizawa, Hashimoto, Denda, Ochi, & So, 2022) and a negative association between fear of COVID-19 and hesitation to vaccinate (vaccine hesitancy) (Willis et al., 2021). At the same time, similar to the relationship of the perceived vulnerability to vaccination behaviour, there is no clear evidence from the COVID-19 studies to argue that greater fears of the disease are associated with COVID-19 vaccination behaviour (Milligan, Hoyt, Gold, Hiserodt, & Otto, 2021). The cognitive assessment of potential threats and personal experience – both are essential (Breakwell & Jaspal, 2020). The studies mention institutional trust as one of the mitigating factors of fear. In particular, if an individual trusts the institutions, that they consider to be in control of the threat, perceived vulnerability and fears may be diminished (Cori et al., 2021; Cori, Bianchi, Cadum, & Anthonj, 2020; Šuriņa, Martinsone, et al., 2021).

Institutional trust can be defined as an individual's assessment of the extent to which they trust the competence and integrity of public institutions (Sonderskov & Dinsen, 2016). Trust in the information, provided by the government, healthcare system and news media, evidence-based information on the hazards of COVID-19, the safety and efficacy of vaccines, and trust in the functioning of these institutions are key factors in the decision to be vaccinated (Al-Amer et al., 2022; Marco-Franco et al., 2021; Paredes, Apaolaza, Marcos, & Hartmann, 2021). According to the SC theory, institutional trust describes the relationship between society and different institutions and is a key factor in overcoming difficulties (Bartscher, Seitz, Siegloch, Slotwinski, & Wehrhöfer, 2021). Its existence or absence (mistrust) has the most direct effect on the public's willingness to act in accordance with the recommendations of the relevant institutions (Li & Sun, 2021). In addition, institutional trust is particularly important in the face of a long-term crisis (Elgar, Stefaniak, & Wohl, 2020). At the same time, according to SC researchers, studies, conducted during the COVID-19 pandemic, highlight the association between individual’s behaviour and interpersonal relationships within homogeneous and heterogeneous groups (Bartscher et al., 2021). In SC theory, one of the factors that characterises the interpersonal relationship is the perceived social support (Ehsan et al., 2019).

Perceived social support is a cognitive perception of an individual, that they have established reliable links with others, and that in a case of need it is possible to get support (Dour et al., 2014). A study conducted in the United Kingdom (Jaspal & Breakwell, 2021) revealed the relationship of the perceived social support with the likelihood of vaccination against COVID-19. In studies of older people, the researchers point to association between perceived social support and vaccination behaviour (Portero de la Cruz & Cebrino, 2020). At the same time, the results of studies show that more pronounced SC at interpersonal level, characterised by closer interpersonal relations, the awareness that in case of need, including illness, there will be the possibility to receive help from the immediate family may be an obstacle to vaccination. The same refers to trust in information, provided by closest people, rather than that provided by the government and healthcare system. It also might be a major obstacle to preventive behaviour, including vaccination against COVID-19 (Bartscher et al., 2021). Similarly, relying on the support of surrounding people can contribute to a biased assessment of the potential risks (perceived vulnerability) and to alleviating fears of potential hazards (Morsut et al., 2021).

Regarding the COVID-19 vaccination behaviour and socio-demographic variables, age, gender and education are the most commonly assessed items. It is important to include these variables in the analysis, as significant differences in personal experience affect cognitive perception and evaluation of information, including perception of risk, perceived vulnerability and institutional trust (Lazarus et al., 2020; Pickles et al., 2021). There are also significant differences in these socio-demographic variables in emotional expression, fear of COVID-19, and behavioural responses, including COVID-19 vaccination behaviour (Lazarus et al., 2020). Regarding the situation in Latvia, the current problem is the low coverage of vaccinations for the elderly, and studies show that COVID-19 can have the worst effects in this age group (Mertoglu et al., 2022). The majority of the researchers point to a positive relationship between the older ages, gender (female), as well as higher level of education and vaccination against COVID-19. However, the results of the systematic reviews carried out during the COVID-19 pandemic (Al-Amer et al., 2022; Lazarus et al., 2020) also reveal the controversial results.

The aim of this study was to identify the factors associated with the COVID-19 vaccination behaviour. Creating an integrative model and including individual motivation for vaccination, institutional trust, perceived social support from relatives and acquaintances, perceived vulnerability, fear of COVID-19 and socio-demographic factors, to explore the mutual relationships between these factors and association with COVID-19 vaccination behaviour. Thus, the study will contribute to the current debate, as well as will supplement the empirical evidence base that could be useful in the future in cases of potentially dangerous virus outbreaks.

## Materials and methods

### The survey

This cross-sectional study and the measurements, used in it, are part of the survey conducted within the framework of the national research programme ‘Challenges and solutions for Latvia’s state and society in an international context (INTERFRAME-LV)’. Data were collected from a representative sample of Latvian residents.

### Variables

#### COVID-19 vaccination behaviour

In order to assess the behaviour of the vaccination, the respondent had to choose one response from the items proposed: ‘I am vaccinated against COVID-19 (one or both doses)’, ‘I have not been vaccinated against COVID-19, but I will certainly undergo vaccination’, ‘I have not been vaccinated against COVID-19 and I think I would rather vaccinate’, ‘I’d rather not undergo vaccination’, ‘I definitely won't get vaccinated’. The responses provided were divided into two groups – vaccinated and unvaccinated. The response option ‘I have been vaccinated against COVID-19 (one or both doses)’ was coded as ‘vaccinated’, while all other variants of responses were coded as ‘unvaccinated’. The statements to assess the behaviour of COVID-19 vaccination were formulated as part of this study.

#### Motivation for vaccination

To evaluate the motivation for vaccination respondents were proposed the following statements: (1) ‘I am vaccinated/will be vaccinated against COVID-19 in order to protect myself from serious infection’, (2) ‘I am vaccinated/will be vaccinated against COVID-19 in order to promote collective immunity, protecting relatives and other people from infection’, (3) ‘COVID-19 vaccination was mandatory to keep a job or to continue studies’. The response scale ranked from 1 to 5 (‘Disagree’ to ‘Agree’). Statements to assess the reason for vaccination were formulated within the scope of this study.

#### Variables of SC theory

For the variables, for which scales were used for assessment, a factor analysis was initially performed, to ensure that a single latent variable with subscales is generated. The Kaiser-Meyer-Olkin criterion was calculated to test the suitability of the data for factor analysis using the Varimax rotation. To calculate the scale values, the arithmetic mean value of the corresponding items was calculated with the SPSS procedure MEAN. The McDonald omega coefficient was calculated for the generated scales: Institutional trust, The perceived social support from the immediate family and The perceived social support from acquaintances.

#### Institutional trust

To evaluate the Institutional trust, the scale from The Multidimensional Social Capital Scale (MSCS V2) (Šuriņa, Ozerska, et al., 2021) was used. Respondents were asked:

Please assess the extent to which you personally trust each of the institutions listed below regarding the provided information and behaviour recommendations during the state of emergency: (1) Government, (2) Latvia’s Saeima, (3) Latvian judicial system, (4) News media, (5) The local government, (6) Police, (7) Preventive care system

The response scale ranged from 1 (‘I do not trust this institution at all’) to 10 (‘I fully trust this institution’). Internal consistency of the scale was (ω = .81).

#### The perceived social support from the immediate family

To evaluate the perceived social support from the immediate family we used the scale from The MSCS V2. Respondents were proposed the following statements: ‘I know I have the closest people to ask for advice’, ‘I know that at a difficult moment my closest people will support me emotionally’, ‘I have an understanding and supportive relationship with the closest people’. The response scale ranged from 1–5 (‘Disagree’ to ‘Agree’). Internal consistency of the scale was (ω = .93)

#### The perceived social support from acquaintances

To evaluate the perceived social support from acquaintances the scale from The MSCS V2 was used. Respondents were proposed the following statements: (1) ‘There are people, among my acquaintances, who would help, if I had financial problems’, (2) ‘There are people, among my acquaintances, who would be able to help with professional advice, if there was such a need’, (3) ‘There are people, among my acquaintances, who, at a difficult moment, would be able to support me emotionally’, (4) ‘There are people, among my acquaintances, who could listen and understand me’. The response scale ranged from 1–to 5 (‘Disagree’ to ‘Agree’). Internal consistency of the scale was (ω = .87)

### Variables of PMT

#### Perceived vulnerability

To evaluate the Perceived vulnerability, respondents were proposed the following statement: ‘I admit the possibility of being infected by COVID-19’. The response scale ranged from 1–5 (‘Disagree’ to ‘Agree’).

#### Fear of COVID 19

To evaluate the fear of COVID-19, respondents were asked: Please assess your level of fear for COVID-19, in the scale 1–10, where 1 means – no fear and 10 – very pronounced fear.

#### Socio-demographic factors

The following socio-demographic data were collected during the study and evaluated as covariates: age and education level: secondary or lower, secondary/professional education and higher education.

#### Data collection procedure

The data were collected from 10 to 22 September 2021. Direct interviews at respondents’ residences were conducted, using the principle of stratified random selection by administrative territorial division. Not less than 100 sampling points were selected randomly from all Latvian populated areas, using the population size in the populated areas as a measure of proportionality. The number of initial sampling points was determined at each populated area. A maximum of 10 interviews were collected at each sampling point. Only one person (respondent) was interviewed in the household. The respondent was chosen according to the principle of the nearest birthday. If the selected respondent was not available, the interviewer attempted to contact him repeatedly. The number of visits to the selected household was three times. If these three contact attempts were unsuccessful, the interviewer selected the respondent in the next household according to the route rules.

#### Participants

The total sample size was *N* = 1017, they all were residents of Latvia, aged 18–75 (*M* = 46.53, *SD* = 16.22). The characteristics of the sample socio-demographic variables are shown in Table 1.

**Table 1.** Descriptive statistics.

Demographic variables	*n* (%)
Age	
18–24	106 (8.4)
25–34	176 (18.7)
35–44	200 (19.6)
45–54	183 (19.1)
55–63	166 (17.4)
64–75	186 (16.8)
Gender	
Male	470 (48.3)
Female	547 (51.7)
Education level	
Primary education	84 (7.9)
Secondary	665 (65.5)
Higher education	268 (26.5)
Marital status	
Married or living with a partner	570 (57.4)
Divorced or not living with husband / wife	133 (13.4)
Single	213 (19.8)
Widow	101 (9.4)
Nationality	
Latvian	604 (59.0)
Russian	337 (33.5)
Other	76 (7.5)
Employment sector	
Public sector (state, municipalities)	172 (17.2)
Private sector	492 (50.0)
Does not work	353 (32.8)
Number of people in the household	
One	234 (22.7)
Two	343 (34.1)
Three	225 (22.3)
Four and more	215 (20.9)
Children under 18 years	
Yes	347 (34.3)
No	670 (65.7)
Region	
Riga	333 (33.4)
Vidzeme	238 (23.5)
Kurzeme	129 (12.4)
Zemgale	172 (17.0)
Latgale	145 (13.7)

Note: *N* = 1017.

### Data analysis

Data analyses were conducted with SPSS version 26.0. At the beginning of the data analysis, the empirical distribution of psychological indicators was tested for the alignment with normal distribution. Since the empirical distribution of all psychological variables did not correspond to the normal distribution, non-parametric methods of data analysis were used in the data analysis. The upper and lower limits were set for psychological variables to identify outliers. Chi-squared test was calculated to establish whether there is an association between socio-demographic variables and COVID-19 vaccination behaviour. The Mann–Whitney Test was calculated to establish whether there is an association between gender and psychological factors included in the model and, whether there is an association between psychological factors and COVID-19 vaccination behaviour. Kruskal Wallis Test was calculated to establish whether there is an association between age, level of education and psychological factors. Spearman's rank correlation coefficient was calculated to establish whether there is an association between the psychological factors included in the model. To clarify the association between the socio-demographic variables, psychological factors and COVID-19 vaccination behaviour included in the model, the Binary Logistic regression analysis, which is suitable when the dependent variable is on a nominal scale (King & Osborne, 2008) with two possible values, was used to establish the factors associated to COVID-19 behaviour. A Binominal logistic regression was performed to ascertain the effect of age, gender, level of education, motive to be vaccinated, institutional trust, the perceived social support from the immediate family, the perceived social support from acquaintances, perceived vulnerability, and fear of COVID-19 on COVID-19 vaccination behaviour.

### Ethics

The study was approved by the Ethics Committee of Research in Rīga Stradiņš University (Register code Nr. 5-1/238/2018).

## Results

### Association between socio-demographic variables and COVID-19 vaccination behaviour

The results of the study show a statistically significant association between age, gender, and educational attainment and COVID-19 vaccination behaviour (see Table 2). Respondents in the age groups from 64 to 75 (54.3%) and from 45 to 54 years (54.1%) are the most vaccinated. On the other hand, the most unvaccinated are in the age groups from 25 to 34 (59.1) and from 18 to 24 (58.5%) years. Women (51.6%) and respondents with higher education (65.7%) are vaccinated more actively.

**Table 2.** Association between demographic variables and COVID-19 vaccination behaviour.

Demographic variables		*n*	Vaccinated, *n* (%)	Not vaccinated, *n (%)*	χ^2^	*p*
Age					12.69 (5)	.026
	18–24	106	44 (41.5)	62 (58.5)		
	25–34	176	72 (40.9)	104 (59.1)		
	35–44	200	89 (44.5)	111 (55.5)		
	45–54	183	99 (54.1)	84 (45.9)		
	55–63	166	73 (44.0)	93 (56.0)		
	64–75	186	101 (54.3)	85 (45.7)		
Gender					9.85 (1)	.001
	Male	470	196 (41.7)	274 (58.3)		
	Female	547	282 (51.6)	265 (48.4)		
Education level					52.77 (2)	.000
	Primary education	84	28 (33.3)	56 (66.7)		
	Secondary	665	274 (41.2)	391 (58.8)		
	Higher education	268	176 (65.7)	92 (34.3)		

Note: *N* = 1017.

### Association between age and psychological factors

Examining the association between the age and psychological factors, a Chi-square test revealed (see Table 3) a statistically significant association between the age and motive for vaccination in order to be able to continue to work and / or study χ^2^ (5) = 34.14, *p* < .001, and motive for vaccination to be protected from severe illness in case of infection χ^2^ (5) = 11.69, *p* = .013. In both cases, respondents in the 64–75 age groups showed a higher motivation for vaccination in order to be able to continue to work and/or study and in order to be protected from severe illness in case of infection.

**Table 3.** Association between age and psychological factors.

Education level	Percentiles	1. Motive to be vaccinated to be protected from severe illness in case of infection	2. Motive to be vaccinated to promote collective immunity, to protect the immediate family and other people from infection	3. Motive to be vaccinated in order to be able to continue to work and/or study	4. Institutional trust	5. Perceived social support from the immediate family	6. Perceived social support from acquaintances	7. Perceived vulnerability	8. Fear of COVID-19
18–24	P25	3.00	2.00	2.00	3.42	4.00	3.75	3.00	2.00
*n* = 106	P50	3.00	3.00	3.00	4.71	4.33	4.00	3.00	5.00
	P75	5.00	4.00	4.25	6.03	5.00	4.75	4.00	7.00
25–34	P25	2.00	2.00	2.00	3.28	4.00	3.50	3.00	2.00
*n* = 176	P50	3.00	3.00	3.00	4.42	4.00	4.00	3.00	5.00
	P75	4.00	4.00	4.00	5.58	5.00	4.50	4.00	7.00
35–44	P25	2.00	2.00	2.00	3.14	4.00	3.50	3.00	3.00
*n* = 200	P50	3.00	3.00	3.00	4.57	4.00	4.00	3.00	5.00
	P75	4.00	4.00	4.00	5.57	5.00	4.25	4.00	8.00
45–54	P25	2.00	2.00	2.00	3.14	4.00	3.50	3.00	3.00
*n* = 183	P50	3.00	3.00	3.00	4.57	4.00	4.00	4.00	5.00
	P75	4.00	4.00	4.00	5.57	5.00	4.50	4.00	8.00
55–63	P25	3.00	2.00	1.00	3.42	4.00	3.50	3.00	3.00
*n* = 166	P50	4.00	3.00	3.00	4.71	4.00	4.00	3.00	6.00
	P75	4.00	4.00	4.00	5.85	5.00	4.13	4.00	8.00
64–75	P25	3.00	2.00	3.00	3.32	4.00	3.25	3.00	2.00
*n* = 186	P50	4.00	3.00	4.00	4.71	4.00	4.00	3.00	5.00
	P75	5.00	4.00	5.00	5.58	5.00	4.50	4.00	8.00
Chi-Square		11.69*	4.95	34.14***	2.06	3.00	6.42	4.91	55.31

Note: *N* = 1017.

*p < .050, **p < .010, ***p < .001.

### Differences between gender and psychological factors

Mann–Whitney U test was run to determine if there were differences between gender and psychological factors. Distribution of psychological factors scores for males and females were not similar in 6 of the 8 psychological factors (see Table 4). The motive for vaccination, to protect oneself from serious illness, scores for females (mean rank = 536.23) were statistically significantly higher than for males (mean rank = 477.31), *U* = 113, *z* = −3.271, *p* = .001. The motive for vaccination, to protect other people and ending a pandemic, scores for females (mean rank = 529.429) were statistically significantly higher than for males (mean rank = 485.25), *U* = 117, *z* = −2.448, *p* = .014. Institutional trust scores for females (mean rank = 528.44) were statistically significantly higher than for males (mean rank = 485.24), *U* = 117, *z* = −2.340, *p* = .019. The perceived social support from the immediate family scores for females (mean rank = 524.50) were statistically significantly higher than for males (mean rank = 451.11), *U* = 101, *z* = −4.215, *p* = .000. The perceived social support from acquaintances scores for females (mean rank = 529.50) were statistically significantly higher than for males (mean rank = 484.11), *U* = 116, *z* = −2.501, *p* = .012. Fear of COVID-19 score for females (mean rank = 526.29) were statistically significantly higher than for males (mean rank = 440.77), *U* = 968, *z* = −4.760, *p* = .000. The motive for vaccination, to continue to work or study, scores for females (mean rank = 520.75) and for male (mean rank = 494.75) were not statistically significantly different, *U* = 121, *z* = −1.419, *p* = .156, and perceived vulnerability for females female (mean rank = 521.38) and for males (mean rank = 494.59) were not statistically significantly different, *U* = 121, *z* = −1.532, *p* = .125.

**Table 4.** Differences between gender and psychological factors.

	Male ( = 470)			Female (*n* = 547)				
	Percentiles							
Variables	P25	P50	P75	P25	P50	P75	Mann–Whitney U	*p*
Motive to be vaccinated to be protected from severe illness in case of infection	2.00	3.00	4.00	3.00	4.00	5.00	113652.50	.001
Motive to be vaccinated to promote collective immunity, to protect the immediate family and other people from infection	2.00	3.00	4.00	2.00	3.00	4.00	117381.50	.014
Motive to be vaccinated in order to be able to continue to work and/or study	2.00	3.00	4.00	2.00	3.00	4.00	121825.000	.156
Institutional trust	3.14	4.42	5.57	3.42	4.71	5.85	117364.00	.019
Perceived social support from the immediate family	4.00	4.00	5.00	4.00	4.00	5.00	101518.50	.000
Perceived social support from acquaintances	3.25	4.00	4.25	3.50	4.00	4.50	116845.00	.012
Perceived vulnerability	3.00	3.00	4.00	3.00	4.00	4.00	121774.00	.125
Fear of COVID-19	2.00	5.00	7.00	3.00	5.00	8.00	96896.00	.000

### Association between education level and psychological factors

Examining the association between educational level and psychological factors, a Chi-square test reveals (see Table 5) a statistically significant association between 6 of the 8 psychological factors. There was no statistically significant association between education level and two variables: fear of COVID-19 and perceived social support from the immediate family.

**Table 5.** Association between education level and psychological factors.

Education level	Percentiles	1. Motive to be vaccinated to be protected from severe illness in case of infection	2. Motive to be vaccinated to promote collective immunity, to protect the immediate family and other people from infection	3. Motive to be vaccinated in order to be able to continue to work and/or study	4. Institutional trust	5. Perceived social support from the immediate family	6. Perceived social support from acquaintances	7. Perceived vulnerability	8. Fear of COVID-19
Primary education *n* = 84	P25	2.00	2.00	1.00	3.42	4.00	3.56	3.00	2.00
	P50	3.00	3.00	2.00	4.35	4.00	4.00	3.00	5.00
	P75	4.00	4.00	3.25	5.53	5.00	4.50	4.00	7.00
Secondary *n* = 665	P25	2.00	2.00	2.00	3.14	4.00	3.50	3.00	3.00
	P50	3.00	3.00	3.00	4.42	4.00	4.00	3.00	5.00
	P75	4.00	4.00	4.00	5.71	5.00	4.25	4.00	8.00
Higher education *n* = 268	P25	3.00	2.00	2.00	3.46	4.00	3.50	3.00	3.00
	P50	4.00	4.00	3.00	5.00	4.33	4.00	4.00	5.00
	P75	5.00	5.00	4.00	6.14	5.00	4.75	4.00	8.00
Chi-Square		24.37***	24.66***	14.09***	11.30**	4.29	6.41*	9.28**	19.87

Notes: *N* = 1017.

*p < .050, ** *p* < .010, *** *p* < .001.

### Differences between psychological factors and COVID-19 vaccination behaviour

Mann–Whitney U test was run to determine if there were differences between psychological factors and COVID-19 vaccination behaviour. Distribution of psychological factors scores for COVID-19 vaccination behaviour were not similar in 7 of the 8 psychological factors (see Table 6). The motive for vaccination, to protect oneself from serious illness, scores for vaccinated (mean rank = 704.38) were statistically significantly higher than for not vaccinated (mean rank = 335.73), *U* = 354, *z* = −29.490, *p* = .000. The motive for vaccination, to protect other people and ending pandemic, scores for vaccinated (mean rank = 695.00) were statistically significantly higher than for not vaccinated (mean rank = 344.05), *U* = 399, *z* = −19.472, *p* = .000. The motive for vaccination, to continue to work or study, scores for vaccinated (mean rank = 592.41) were statistically significantly higher than for not vaccinated (mean rank = 433.95), *U* = 884, *z* = −8.817, *p* = .000. Institutional trust scores for vaccinated (mean rank = 589.84) were statistically significantly higher than for not vaccinated (mean rank = 436.51), *U* = 897, *z* = −8.315, *p* = .000. The perceived social support from the immediate family scores for vaccinated (mean rank = 512.83) were statistically significantly higher than for not vaccinated (mean rank = 469.92), *U* = 109, *z* = −2.469, *p* = .014. The perceived social support from acquaintances scores for vaccinated (mean rank = 536.97) were statistically significantly higher than for not vaccinated (mean rank = 483.30), *U* = 114, *z* = −2.960, *p* = .003. Fear of COVID-19 scores for vaccinated (mean rank = 540.63) were statistically significantly higher than for not vaccinated (mean rank = 437.91), *U* = 931, *z* = −5.730, *p* = .000. Perceived vulnerability scores for vaccinated (mean rank = 514.13) and for not vaccinated (mean rank = 504.45) were not statistically significantly different, *U* = 121, *z* = −1.419, *p* = .156.

**Table 6.** Differences between psychological factors and COVID-19 vaccination behaviour.

	Vaccinated (*n* = 478)			Not vaccinated (*n* = 539)				
Variables	P25	P50	P75	P25	P50	P75	Mann-Whitney U	*p*
2. Motive to be vaccinated to be protected from severe illness in case of infection	4.00	4.00	5.00	2.00	3.00	3.00	35430.00	.000
3. Motive to be vaccinated to promote collective immunity, to protect the immediate family and other people from infection	3.75	4.00	5.00	2.00	2.00	3.00	39911.50	.000
4. Motive to be vaccinated in order to be able to continue to work and/or study	2.00	3.00	5.00	1.00	3.00	3.00	88472.00	.000
5. Institutional trust	3.78	5.00	6.28	2.85	4.00	5.28	89750.00	.000
6. Perceived social support from the immediate family	4.00	4.33	5.00	4.00	4.00	5.00	109356.50	.014
7. Perceived social support from acquaintances	3.50	4.00	4.50	3.25	4.00	4.25	114971.00	.003
8. Perceived vulnerability	2.75	3.00	3.50	2.75	3.00	3.50	126368.50	.595
9. Fear of COVID-19	3.00	6.00	8.00	2.00	4.00	7.00	93173.50	.000

### Association between psychological factors

Examining the association between psychological factors (see Table 7), we found a statistically significant weak or very weak association between most psychological factors. There was a statistically significant, strong association between motive for vaccination to be protected from severe illness in the case of infection and motive for promoting collective immunity, protect the immediate family and other people from infection, *r* (1015) = .784, *p* < .001. We found also statistically significant, strong association between perceived social support from the immediate family and perceived social support from acquaintances, *r* (978) = .662, *p* < .001. The results of the study do not reveal a statistically significant association between perceived social support from the immediate family and fear of COVID-19, perceived social support from the immediate family and motive for vaccination in order to be able to continue working and/or study.

**Table 7.** Association between psychological factors.

Variables	1.	2.	3.	4.	5.	6.	7.	8.
1. Motive to be vaccinated to be protected from severe illness in case of infection	–							
2. Motive to be vaccinated to promote collective immunity, to protect the immediate family and other people from infection	.784**	–						
3. Motive to be vaccinated in order to be able to continue to work and/or study	.299**	.340**	–					
4. Institutional trust	.326**	.290**	.066*	–				
5. Perceived social support from the immediate family	.146**	.122*	.009	.182**	–			
6. Perceived social support from acquaintances	.104**	.094**	.088*	.105*	.662**	–		
7. Perceived vulnerability	.137**	.137**	.062*	.064*	.115**	.091**	–	
8. Fear of COVID-19	.275**	.243**	.094**	.178**	.024	.083**	.142**	–

Notes: Spearman's rank correlation coefficient. *N* = 1017.

*p < .050, ** *p* < .010, *** *p* < .001.

### Association between socio-demographic, psychological factors and COVID-19 vaccination behaviour

A Binominal logistic regression was performed to ascertain the effect of age, gender, level of education, motive to be vaccinated, institutional trust, the perceived social support from the immediate family, the perceived social support from acquaintances, perceived vulnerability, and fear of COVID-19 on COVID-19 vaccination behaviour. Socio-demographic variables were inserted in the first step, psychological factors added in the second step. Linearity of the continuous variables, with respect to the logit of dependent variable, was assessed via the Box-Tidwell (2001) procedure (Li, Martin, & Morris, 2001). A Bonferroni correction was applied, using all twenty-two variables in the model, resulting in statistical significance being accepted when *p* < .00277 (Tabachnick & Fidell, 2014). Based on this assessment, all continuous independent variables were found to be linearly related to the logit of the dependent variable. There were 31 standardised residuals with a value interval from 2.612 up to 8.814 and from −5.040 up to −2.649. These participants were excluded from further analysis. The logistic regression model was statistically significant χ^2^ (11) = 684.27, *p* < .001. The model explained 70.9% (Nagelkerke *R^2^*) of the variance in vaccination behaviour and correctly classified 86.7% of cases. Sensitivity was 86.9%, specificity was 86.5%, positive predictive value was 85.71%, and negative predictive value was 87.64%. The area under the ROC curve was .940 (95% CI, .925 to.954) which is an outstanding discrimination (Hosmer, Lemeshow, & Sturdivant, 2013). Of the 11 independent variables, included in the model, 6 were statistically significant to explore COVID-19 vaccination behaviour. They are the following: level of education, reason for vaccination to protect oneself from illness, to promote collective immunity, to protect one’s relatives and others from infections, to be able to continue working and/or studying, institutional trust, and perceived social support from the immediate family (see Table 8).

**Table 8.** Association between socio-demographic, psychological factors and COVID-19 vaccination behaviour.

			95.0% Confidence interval for B			
Variable	B	S.E.	OR	Lower Bound	Upper Bound	*p*
Age	–	–	–	–	–	.136
18–24	-.732	.472	.481	.191	1.212	.121
25–34	-.352	.374	.703	.338	1.463	.346
35–44	-.444	.369	.641	.311	1.321	.228
45–54	.081	.363	1.084	.532	2.209	.824
55–63	-.794	.363	.473	.232	.963	.039
64–75 (RC)	–	–	–	–	–	–
Gender	–	–	–	–	–	.815
Male	0.52	0.22	1.053	.682	1.627	
Female (RC)	–	–	–	–	–	–
Education level	–	–	–	–	–	.000
Primary education	-.833	.440	.435	.184	1.030	.058
Secondary	−1.267	.268	.282	.167	.476	.000
Higher education (RC)	–	–	–	–	–	–
Motive to be vaccinated to be protected from severe illness in case of infection	1.472	.153	4.357	3.229	5.878	.000
Motive to be vaccinated to promote collective immunity, to protect the immediate family and other people from infection	.943	.129	2.560	1.993	3.311	.000
Motive to be vaccinated in order to be able to continue to work and/or study	.373	.096	1.452	1.203	1.754	.000
Institutional trust	.181	.067	1.198	1.050	1.367	.007
Perceived social support from the immediate family	-.414	.201	.661	.445	.980	.039
Perceived social support from acquaintances	.015	.189	1.015	.701	1.470	.937
Perceived vulnerability	-.209	.119	.811	.643	1.024	.078
Fear of COVID-19	-.027	.042	.973	.896	1.056	.514

RC: reference category; B: unstandardised coefficient; SE: standard error; Exp(B): exponentiated regression coefficient.

When comparing respondents with primary education to respondents with higher education, respondents with lower education are more likely to be unvaccinated, but differences are not statistically significant (OR = 2.298, 95% CI [0.970–5.436], *p* = .058). On the other hand, when comparing respondents with secondary education to respondents with higher education, respondents with secondary education are more likely to be unvaccinated, and this difference is statistically significant (OR = 3.546, 95% CI [2.100–5.988], *p* < .001). The results of the study also reveal three individual vaccination motives statistically significantly associated with vaccination behaviour. A significant motive is to protect oneself from serious illness (OR = 4.357, 95% CI [3.229–5.878], *p* < .001). Another significant motive for vaccination is to promote collective immunity, to protect close relatives and others from infection (OR = 2.560, 95% CI [1.993–3.311], *p* < .001). Third significant motive for vaccination is to keep the job or continue studying (OR = 1.452, 95% CI [1.203–1.754], *p* < .001). Thus, these motives increase the likelihood of people being vaccinated. The higher the institutional confidence rates (OR = 1.198, 95% CI [1.050–1.367], *p* =.007), the higher the probability of being vaccinated. On the other hand, the higher the perceived social support from the immediate family, the higher the probability of being unvaccinated (OR = 1.512, 95% CI [1.020–2.247], *p* = .039).

Age, gender, perceived social support from acquaintances, perceived vulnerability, and fear of COVID-19, in the sample, did not appear to be statistically significant associated with COVID-19 vaccination behaviour.

## Discussion

Based on the theory of SC and PMT, an integrative model was developed in this study, which allows for the explanation of factors associated with COVID-19 vaccination behaviour. The integrative model included factors such as institutional trust, perceived social support from relatives and acquaintances, perceived vulnerability, fear of COVID-19, individual vaccination motives, education level and age.

As the results of our study reveal, the level of education is the main sociodemographic factor associated with COVID-19 vaccination behaviour. While initially, when testing the association between age, gender and educational levels and vaccination behaviour, all these socio-demographic variables were statistically significant. After inclusion of these variables in the overall regression analysis model, only the level of education remained statistically relevant to COVID-19 vaccination behaviour. This indicates the importance of the critical selection, perception, and understanding of information on the safety and efficacy of COVID-19 vaccines. Similar results appear also in other studies carried out during the pandemic (Lazarus et al., 2020), However, the results of the studies are ambiguous (Al-Amer et al., 2022; Lazarus et al., 2020). This could be explained by the interaction between factors, such as age, gender, education level, personal experiences that affect perception and interpretation of information, trust, perceived vulnerability, emotional aspects, including fear of COVID-19, motivation for vaccination and subsequent behaviour as a result (Lazarus et al., 2020).

As in other studies, the results of this study also revealed that three vaccination motives included in the model are associated with vaccination behaviour. The motive for vaccination to protect oneself from serious illness shows awareness of the risk of illness and vaccination as an effective form of protection (Reinders et al., 2020; Yaqub, Castle-Clarke, Sevdalis, & Chataway, 2014). The study authors highlight the relevance of this motive to knowledge, attitude to vaccines and vaccination and trust in sources that disseminate evidence-based, science-based information (Al-Amer et al., 2022; Yaqub et al., 2014). The motive for vaccination to protect other people and ending a pandemic is very important, because of the dangers of COVID-19 for individual population groups, such as elderly people, people with chronic diseases, with weakened immunity, etc. (Giubilini et al., 2020). Also in our study, the results revealed a strong relationship between motive for vaccination to protect oneself from serious illness and motive to protect other people. As researchers show, the motive to protect other people can be an important factor in deciding to be vaccinated, even if the individual does not see the need to protect themselves (Machida et al., 2021; Rieger, 2020). Such behaviour can be interpreted as altruistic behaviour or solidarity, as highlighted in the SC theory (Ferwana, Varshney, & Patel, 2021; Rieger, 2020).

In a number of countries, vaccination is a requirement of different institutions and employers for employees in certain professions/students in order to keep their jobs or continue studying, without endangering their health and the health of others (Palm et al., 2021; Riva et al., 2021). The results of our study also show that compulsory vaccination, so that person can continue to work or study, is related to COVID-19 vaccination behaviour. Although compulsory vaccination is a way of promoting collective immunity, researchers draw attention to the fact that this is not a long-term solution, since compulsory vaccination leads to resistance and may lead to negative attitude to vaccination in general, which may have far-reaching consequences (Marco-Franco et al., 2021). Researchers highlight the importance of communication, education and clarification to promote knowledge and awareness of the hazards of the virus, safety and efficacy of vaccines (Palm et al., 2021; Riva et al., 2021). The more educated and knowledgeable the public is the more positive is the attitude toward vaccination and the higher COVID-19 vaccination scope (Riva et al., 2021).

Like other studies (Al-Amer et al., 2022; Lazarus et al., 2021), our study finds that institutional trust is related to COVID-19 vaccination behaviour. Institutional trust is also related to individual vaccination motives and other factors included in the model. Trust is fundamentally important for public involvement in tackling common challenges. The situation in Latvia clearly highlights the association between the historically low confidence rates of the Latvian public (Eurobarometer, 2020) and the low and slow-growing vaccination coverage (NVD, 2022). Looking at institutional trust, a factor in SC, the researchers point to the association between confidence in public institutions, public sentiment and the long-term benefits of society (Ferwana et al., 2021). Relying on information provided by national authorities and mass media, supported by evidence and acting in accordance with recommendations, including vaccination against COVID-19, the benefit is gained not only by individuals protecting themselves from the disease, but also by the general public, as anyone who has been vaccinated increases the total vaccination scope (Ferwana et al., 2021). The researchers point out that when the crisis is sustained, public confidence is diminishing. It means that there will also be a reduction in readiness to act in accordance with the recommendations of the government and other institutions, therefore, clear, direct communication is important from the government and other institutions, which does not contradict action, thereby strengthening confidence in public institutions (Elgar et al., 2020; Pitas & Ehmer, 2020).

Looking at the association of COVID-19 infection prevalence and prevention behaviour, SC researchers reveal a negative relationship between bonding SC and prevention behaviour (Elgar et al., 2020). Similar results are also revealed in our study. Perceived social support from close relatives is negatively related to COVID-19 vaccination behaviour. Namely, the higher the perceived social support rates, the less likely that the person will be vaccinated. SC researchers explain this relation in the sense that closer relations within homogenous groups, information exchange and trust in this information, as well as confidence that assistance will be possible, if necessary, exclude, to some extent, the adoption of external information and trust in this information. It can be classified, in this case, as institutional trust and, consequently, the lack of involvement in the recommended behaviour (Elgar et al., 2020; Ferwana et al., 2021). Consequently, the issue of government, healthcare professionals and mass media communication channels and types targeting all groups of society is raised, with particular focus on potentially socio-demographic homogenous groups with, to certain extent, closed information space (Ferwana et al., 2021; Thompson et al., 2021). On the other hand, perceived social support from acquaintances was not identified as a statistically significant factor. It shows that relationships and, if necessary, potential support from acquaintances is not essential to influence the individual's decision to be vaccinated.

Unlike other studies (Al-Amer et al., 2022), the perceived vulnerability in our study was not identified as a factor related to COVID-19 vaccination behaviour. As part of the PMT, risk assessment and the perceived vulnerability that forms it are considered to be the most significant factors for anticipating preventive behaviour (Chen, Lin, Chang, Chou, & Yen, 2021). However, it is important to take a number of aspects into account here. Firstly, if a person is vaccinated, they will no longer feel vulnerable. Secondly, as mentioned above, the threat assessment includes the seriousness of the perceived threat and perceived vulnerability (Jaspal, Fino, & Breakwell, 2020). Our study assessed the perceived vulnerability that reflects an individual's assessment of the risk of illness, but not an assessment of the severity of the disease. As Van Der Pligt with colleagues (Van Der Pligt, 1998) has already pointed out, the perceived vulnerability, in its content, may be an insufficiently valid measure to assess the relationship of threats and preventive behaviour. Thirdly, as researchers point out, when assessing the relationship of perceived vulnerability to preventive behaviour, dispositional optimism should be taken into account, which may significantly undermine the assessment of potential hazards (Kapoor & Singhal, 2021). Fourthly, at the end of 2019, when the first cases of COVID-19 were detected, there was no information on this disease (Chen & Yu, 2020). In many parts of the world, governments responded very cautiously introducing strict safety measures and limiting the spread of the disease (Meunier, 2020). Knowledge and understanding of the virus gradually accumulated and it was clear that COVID-19 had high transmission, but is dangerous for individual groups of society (Moline et al., 2021). At the same time a variety of false news and conspiracy theories about COVID-19 spread (Simione et al., 2021; Šuriņa, Martinsone, et al., 2021). All this obviously affects the assessment of the severity of the disease and of potential for infection. Fifthly, threat assessment and perceived vulnerability may relate to the experience of the disease (Breakwell & Jaspal, 2020). Given that COVID-19 may be particularly severe for individual groups of society, but in most cases the public has mild or moderate symptoms, a large part of the population is convinced that the disease is not actually so dangerous and that the risk of infection is not too high (Breakwell & Jaspal, 2020). These arguments largely explain why the perceived vulnerability in the results of this study, as well as in other studies (Hromatko et al., 2021; Lau et al., 2010) are not related with vaccination behaviour. Within PMT, the cognitive assessment of potential threats is closely linked to the emotional assessment of potential threats (Adunlin et al., 2020; Tong et al., 2021). Namely, the cognitive assessment of the potential for infection is associated with an appropriate emotional response, which the results of our study also confirm. Like perceived vulnerability, fear of COVID-19 was not identified as a factor related with vaccination behaviour. When examining the individual association between psychological factors and vaccination behaviour, fear of COVID-19 was associated with vaccination behaviour. However, when included in the overall model, it was no longer statistically significant. Here one should take into account the above mentioned regarding the public's overall understanding of the risks of the virus, past experience with the virus as well as the interaction of other factors included in the model (Breakwell & Jaspal, 2020).

As the strengths of our study, we would like to highlight, firstly, the integrative model within SC and PMT, which makes it possible to explain the behaviour of vaccination in the light of the interaction between psycho-emotional, cognitive and socio-demographic factors. Secondly, the nationally representative sample comprises different socio-demographic groups, which allows the results of our study to extend to all Latvian residents and to be applied in a similar context. Thirdly, the study used an instrument developed and tested in the cultural environment of Latvia: MSCS V2 (Šuriņa, Martinsone, et al., 2021) to assess institutional trust and perceived social support from relatives and acquaintances.

The study also has several limitations. Since this study, with the measurements used in it, was part of a wider survey, we could include a certain number of items in the overall survey. As a result, we used one item to assess the fear of COVID-19, instead of using internationally tested measurement tool The Fear of COVID-19 scale (Ahorsu, Lin, & Pakpour, 2020). Similarly, we used one item to assess perceived vulnerability, but not internationally tested measurement tool The COVID-19 Own Risk Appraisal Scale (CORAS) (Jaspal et al., 2020).

Referring to the current discussion in the scientific literature, the use of regression analysis is another limitation. Although regression analyses are commonly used today for selecting determinants to target in behaviour change interventions, regression analyses may not be suitable for this purpose (see Peters & Crutzen, 2021). Bivariate analyses can provide insight into the associations between multiple determinants and the target behaviour. Being associated with behaviour is a prerequisite for a determinant to be selected. However, these bivariate analyses need to be combined with the univariate distribution of each determinant, and there are freely accessible tools available to do so (Crutzen, Peters, & Noijen, 2017; Peters & Crutzen, 2021)

The model developed under SC and PMT contributes to the understanding of the factors for anticipating COVID-19 vaccination behaviour. From the results of the study, we can conclude that knowledge and confidence in evidence-based information on the hazards of COVID-19 and the safety and efficacy of vaccines, provided by official sources of information, is an essential factor in the decision to protect oneself from serious illness, or in order to promote collective immunity and protect other people. The results of the study also revealed that an essential motive for vaccination is the requirement of employers. On the other hand, the belief that support can be obtained, if necessary, which, within the framework of the SC theory, shows close relations within homogenous groups, may be an obstacle for a person to become vaccinated.

It is important to continue to identify promoting and delaying factors for vaccination, to study in detail the interaction of individual motives with social, cognitive, and emotional factors, as well as to assess changes in the interaction of these factors over time. The experience and knowledge accumulated can be useful in cases of potential virus outbreaks in the future (Behl et al., 2022; Wan et al., 2022).

## Data Availability

The raw data available: https://doi.org/10.48510/FK2/ZDNPH3.
